# SLCO4A1-AS1 promotes colorectal tumourigenesis by regulating Cdk2/c-Myc signalling

**DOI:** 10.1186/s12929-022-00789-z

**Published:** 2022-01-17

**Authors:** Jia Zhang, Kaisa Cui, Liuying Huang, Fan Yang, Shengbai Sun, Zehua Bian, Xue Wang, Chaoqun Li, Yuan Yin, Shengling Huang, Leyuan Zhou, Bojian Fei, Zhaohui Huang

**Affiliations:** 1 Wuxi Cancer Institute, Affiliated Hospital of Jiangnan University, 200 Hui He Road, Wuxi, 214062 Jiangsu China; 2 Laboratory of Cancer Epigenetics, Wuxi School of Medicine, Jiangnan University, Wuxi, 214122 Jiangsu China; 3 Institutes of Biomedical Sciences and Shanghai Cancer Center, Shanghai Medical College, Fudan University, Shanghai, 200032 China; 4 Department of Radiation Oncology, Affiliated Hospital of Jiangnan University, Wuxi, 214122 China; 5 Department of Surgical Oncology, Affiliated Hospital of Jiangnan University, Wuxi, 214122 Jiangsu China

**Keywords:** Colorectal cancer, Long non-coding RNA, SLCO4A1-AS1, Cdk2, c-Myc

## Abstract

**Background:**

SLCO4A1-AS1 was found to be upregulated in several cancer types, including colorectal cancer (CRC). However, the detailed roles of SLCO4A1-AS1 in CRC remain to be elucidated. Therefore, we investigated the functions, mechanism, and clinical significance of SLCO4A1-AS1 in colorectal tumourigenesis.

**Methods:**

We measured the expression of SLCO4A1-AS1 in CRC tissues using qRT-PCR and determined its correlation with patient prognosis. Promoter methylation analyses were used to assess the methylation status of SLCO4A1-AS1. Gain- and loss-of-function assays were used to evaluate the effects of SLCO4A1-AS1 on CRC growth in vitro and in vivo. RNA pull-down, RNA immunoprecipitation, RNA-seq, luciferase reporter and immunohistochemistry assays were performed to identify the molecular mechanism of SLCO4A1-AS1 in CRC.

**Results:**

SLCO4A1-AS1 was frequently upregulated in CRC tissues based on multiple CRC cohorts and was associated with poor prognoses. Aberrant overexpression of SLCO4A1-AS1 in CRC is partly attributed to the DNA hypomethylation of its promoter. Ectopic SLCO4A1-AS1 expression promoted CRC cell growth, whereas SLCO4A1-AS1 knockdown repressed CRC proliferation both in vitro and in vivo. Mechanistic investigations revealed that SLCO4A1-AS1 functions as a molecular scaffold to strengthen the interaction between Hsp90 and Cdk2, promoting the protein stability of Cdk2. The SLCO4A1-AS1-induced increase in Cdk2 levels activates the c-Myc signalling pathway by promoting the phosphorylation of c-Myc at Ser62, resulting in increased tumour growth.

**Conclusions:**

Our data demonstrate that SLCO4A1-AS1 acts as an oncogene in CRC by regulating the Hsp90/Cdk2/c-Myc axis, supporting SLCO4A1-AS1 as a potential therapeutic target and prognostic factor for CRC.

**Supplementary Information:**

The online version contains supplementary material available at 10.1186/s12929-022-00789-z.

## Background

More than 1.9 million new colorectal cancer (CRC) cases and approximately 100,000 deaths were estimated in 2020, ranking the third in terms of incidence and the second in terms of mortality representing approximately one in ten cancer cases and deaths worldwide [1]. The pathogenesis of CRC involves multiple factors, including environmental, genetic, and epigenetic variables, and its detailed molecular mechanisms remain unclear [2]. During the past decade, long noncoding RNAs (lncRNAs) have been linked to cancer occurrence and metastasis by acting as either oncogenes or tumour suppressors [3, 4]. These lncRNAs are generally endogenous, no shorter than 200 nucleotides in length and have limited or no protein-coding capacity [5]. Dysregulation of lncRNAs has been implicated in the development and progression of almost all types of tumours, including CRC.

CRC exhibits abnormal expression patterns of lncRNAs, and some lncRNAs, including MEG3[6], SLCC1 [7], H19 [8], FEZF1-AS1 [9], UCA1 [10], and LINC00152 [11], have been reported to regulate colorectal tumourigenesis and progression by targeting tumour-related signalling pathways. We previously identified several lncRNAs with important roles in CRC and demonstrated that lncRNAs appear to be promising cancer biomarkers [9–15]. For example, we revealed that FEZF1-AS1 promote CRC growth and metastasis by regulating STAT3 signalling and glycolysis [9]. These data demonstrate the key roles of lncRNAs in the complicated pathogenesis of CRC.

SLCO4A1 antisense RNA 1 (SLCO4A1-AS1) is located on the opposite strand of SLCO4A1 on chromosome 20, which is significantly overexpressed in multiple cancer types, including CRC [16–21]. Limited studies have reported that SLCO4A1-AS1 could promote tumourigenesis and metastasis via different mechanisms, particularly by regulating different microRNAs (miRNAs) [16–21].

In this study, we confirmed the aberrant overexpression and prognostic role of SLCO4A1-AS1 in CRC using multiple cohorts and revealed that decreased DNA methylation levels in the promoter of SLCO4A1-AS1 account for, at least partly, the aberrant overexpression of SLCO4A1-AS1 in CRC. Functional analyses revealed that SLCO4A1-AS1 markedly promotes tumour growth both in vitro and in vivo. Further mechanistic investigations revealed that SLCO4A1-AS1 acts as a molecular scaffold to strengthen the interaction between Hsp90 and Cdk2 and subsequently promotes Cdk2 protein stability in CRC cells. Finally, the SLCO4A1-AS1-induced increase in Cdk2 levels activates the c-Myc signalling pathway by promoting the phosphorylation of c-Myc at Ser62. Collectively, these data highlight a novel regulatory role of SLCO4A1-AS1 in c-Myc signalling via the Hsp90-Cdk2 axis in CRC, suggesting that SLCO4A1-AS1 represents a potential therapeutic target and prognostic factor for CRC.

## Methods

### Patient information and cell lines

Two CRC cohorts, comprising 109 (CRC cohort 1) and 158 (CRC cohort 2) human primary CRC tissues and their adjacent noncancerous tissues (NCTs) were collected from Fudan University Shanghai Cancer Center with informed consent and Affiliated Hospital of Jiangnan University, respectively. The detailed information of patients was listed in Additional file 1: Table S1.

CRC cell lines (HT29, LoVo, HCT116, SW620, and SW480) and the normal colon epithelial cell line NCM460 were purchased from the American Type Culture Collection (ATCC). LoVo and SW620 cells were cultured in F12K and RPMI 1640, respectively, whereas other cells were maintained in DMEM. All media were supplemented with 10% fetal bovine serum and incubated at 37 °C and 5% CO_2_. These lines were tested for mycoplasma contamination before use to ensure that they were mycoplasma-free.

### Cell transfection and constructions of stable cell lines

Small interfering RNAs (siRNAs) against human SLCO4A1-AS1 (siSLC, 5′-GTTTCTGAGAACTGACATA-3′) were purchased from RiboBio (China). The pLenti-EF1a-EGFP-F2A-Puro-CMV-MCS (oeCtrl), pLenti-EF1a-EGFP-F2A-Puro-CMV-SLCO4A1-AS1 (oeSLC), pLKD-CMV-G&PR-U6-shRNA (shCtrl), and pLKD-CMV-G&PR-U6-shSLCO4A1-AS1 (shSLC) plasmids were purchased from OBiO (China). Plasmids or siRNAs were transfected into cells using the LipoFiter transfection reagent (HanBio, China). The generation of lentiviral particles and the constructions of stable cell lines were conducted as we previously described [9].

### RNA extraction and quantitative RT-PCR analyses

Total RNA was extracted from tissues or cells using an RNA Isolater (Vazyme, China) under RNase-free conditions according to the manufacturer’s protocol. Cytoplasmic and nuclear RNA were separated to analyze RNA subcellular localization according to the instructions of a PARIS™ Kit (Ambion, USA). RNA was reverse transcribed into complementary DNA (cDNA) using a HiFiScript cDNA Synthesis Kit (CWBIO, China). Quantitative RT-PCR (qRT-PCR) was performed using UltraSYBR Mixture (CWBIO), with ACTB as an internal control and U6 small nuclear RNA as a nuclear endogenous control. The primers used for qRT-PCR were listed in Additional file 2: Table S2.

### Promoter methylation analyses

Genomic DNA was extracted from cancer cells or human leukocytes using a Genomic DNA Mini Preparation Kit (Beyotime, China) and then bisulfite-modified using an EpiJET Bisulfite Conversion Kit (Thermo Fisher, USA). To determine the methylation status of each CpG site, the resulting modified DNA was amplified with bisulfite sequencing PCR (BSP) primers, and the PCR products were then inserted into a T-vector for Sanger sequencing. To perform methylation-specific PCR (MSP) analyses, bisulfite-treated DNA was amplified using methylation-specific primers, and the PCR products were subjected to electrophoresis using 2% agarose gels. BSP and MSP primers were designed using MethPrimer (http://www.urogene.org/cgi-bin/methprimer2/MethPrimer.cgi) [22] and listed in Additional file 2: Table S2. NCM460 and LoVo Cells were treated with 10 μmol/L 5-aza-dC (Beyotime, inhibitor of DNA Methyltransferase) for 72 h, and then subjected to the quantitation of SLCO4A1-AS1 by qRT-PCR.

### Cell proliferation and colony formation assays

Cell Counting Kit 8 (CCK8, Dojindo, Japan) and colony formation assays were performed to evaluate cell proliferation abilities as we previously described [9].

### Cell cycle analysis

CRC cells were synchronized by serum starvation for 24 h and were then released into the cell cycle with 20% fetal bovine serum. Cells were fixed in chilled 75% ethanol at 4 °C overnight and subsequently resuspended in 800 μl of phosphate-buffered saline. Next, the fixed cells were stained with propidium iodide (PI, CWBIO) and assessed by a flow cytometer system (BD Biosciences, USA) to analyze the percentage of cells in different phases of the cell cycle.

### Tumourigenesis in nude mice

To assess the in vivo effect of SLCO4A1-AS1 on tumourigenesis, a total of 2 × 10^6^ HCT116 (SW620) cells stably overexpressing SLCO4A1-AS1 (shSLC) or the control vector were subcutaneously injected into the flanks of 5-week-old athymic nude BALB/c mice to establish a CRC xenograft model. When a tumour was palpable, its volume was measured every two days and was calculated according to the formula: volume (mm^3^) = longer diameter × shorter diameter^2^ × 0.5. All protocols concerning animals were approved by the Clinical Research Ethics Committees of Jiangnan University.

### Western blotting and co-immunoprecipitation (co-IP)

Whole-cell lysates were prepared using RIPA buffer containing protease inhibitors (Beyotime). The separation of nuclear and cytoplasmic protein fractions was performed using a Nuclear and Cytoplasmic Protein Extraction Kit (Beyotime). Extracted proteins were separated by SDS-PAGE and transferred to a PVDF membrane. The membrane was blocked with 5% nonfat milk, incubated with primary antibodies at 4 °C overnight, and analyzed by immunoblotting using HRP-conjugated secondary antibodies. Co-IP assays were performed as previously described [23]. Antibodies used in western blotting and co-IP assays were purchased from the following manufacturers: Proteintech (Cdk2, Hsp90, and c-Myc), Abmart (Phospho-c-Myc (S62), Histone H3.1, Myc-Tag, and Flag-tag), ABclonal (β-actin), Absin (GAPDH), and CST (HA-Tag). An anti-β-actin antibody was used as a control for whole-cell lysates. Histone H3.1 and GAPDH antibodies were used as markers of nuclear and cytoplasmic protein fractions, respectively. Normal rabbit IgG or mouse IgG antibody was used as an isotype control for co-IP arrays.

### RNA pull-down and mass spectrometry analyses

RNA pull-down assay was performed as we previously described [9]. Detailed information regarding the primers used for in vitro transcription is depicted in Additional file 2: Table S2. The retrieved proteins in RNA pull-down assays were separated on SDS-PAGE gels for protein mass spectrometry and western blot analyses.

### RNA immunoprecipitation (RIP)

RIP was performed using a Magna RIP Kit (Merck, USA) according to the manufacturer's instructions. Briefly, cells were lysed in RIP lysis buffer supplemented with RNase and protease inhibitors, followed by the addition of magnetic beads coated with a specific antibody to enrich RNA–protein complexes. Precipitated RNAs were then eluted and subjected to qRT-PCR analyses.

### RNA-seq and bioinformatics analyses

Online RNA-seq and DNA methylation data were downloaded from The Cancer Genome Atlas (TCGA) (http://cancergenome.nih.gov/). DNA methylation data of cancer cell lines were collected from Cancer Cell Line Encyclopedia (CCLE) (http://www.broadinstitute.org/ccle/). Four independent microarray datasets (GSE9348, GSE21510, GSE23878, and GSE33113) were available in GEO (http://www.ncbi.nlm.nih.gov/geo). RNA-seq assays were performed to measure the mRNA expression profiles of SLCO4A1-AS1-silenced HT29 cells or SLCO4A1-AS1-overexpressing LoVo cells as well as their control cells at GENEWIZ (China) using HiSeq3000 (Illumina, USA). Gene set enrichment analysis (GSEA) was launched to identify genes of statistical difference by using GSEA v3 software (http://www.brodinstitute.org/gsea/index.jsp) [24]. Differential expression genes (DEGs, |logFC|≧0.5) were clustered and visualized using the pheatmap R package. The clusterProfiler R package was used to identify and visualize the enrichment results of Gene Ontology (GO) and Kyoto Encyclopedia of Genes and Genomes (KEGG) pathways of DEGs [25].

### Luciferase reporter assay

A minimal promoter (TATA-box) and four tandem repeats of canonical c-Myc E-boxes (CACGTG) were synthesized and inserted upstream of the firefly luciferase reporter gene (luc +) in pGL3-Basic vector (Promega, USA), which was used to evaluate the transcription activation levels of c-Myc. The constructed plasmids and the pRL-TK plasmids (Renilla luciferase) were cotransfected into CRC cells. Twenty-four hours after transfection, the cells were harvested and subjected to luciferase activity analyses using the Dual-Luciferase® Reporter Assay System (Beyotime). The primers used for constructs were listed in Additional file 2: Table S2.

### Fluorescent in situ hybridization (FISH) and immunohistochemistry (IHC) staining

The fluorescent in situ detection of SLCO4A1-AS1 was performed on HCT116 cells and 4 µm fresh frozen tissue slides according to the manufacture’s protocol provided by Ribo™ Fluorescent In Situ Hybridization Kit (RiboBio). IHC staining was performed on 4-μm sections of the tissue microarrays constructed using paraffin-embedded CRC tissue samples with primary antibodies for Cdk2 (1:50, Proteintech) or Phospho-c-Myc (62) (p-62 c-Myc, 1:100, Abmart). Next, the expression levels of Cdk2 and p-62 c-Myc were quantified using the GTVision™ Detection System (Gene Tech, China). The total Cdk2 and p-62 c-Myc immunostaining scores were evaluated based on the percentage of positively stained cells and the staining intensity. The percent positivity was scored on a scale of 0–4 as follows: 0 (0%), 1 (1–25%); 2 (26–50%; 3(51–75%) and 4 (> 75%). The staining intensity was established from 0 to 3, with 0 for no staining, 1 for weakly stained, 2 for moderately stained, and 3 for strongly stained. Both the percent positivity of cells and the staining intensity were evaluated under double-blind conditions by two independent pathologists.

### Statistical analyses

Statistical analyses were performed using SPSS 23.0 (IBM, USA) or R (version 4.0.3). GraphPad Prism 8.0 software and R software were utilized to create diagrams. The most appropriate cutoff value for SLCO4A1-AS1 was obtained by generating receiver operating characteristic (ROC) curves in our CRC cohorts. The Pearson χ^2^ test was used to analyze the association between SLCO4A1-AS1 expression and the clinicopathologic features of CRC patients. The Kaplan–Meier method was used to estimate overall survival (OS) and disease-free survival (DFS) and the log-rank test was used to evaluate the differences in survival rates. A Cox proportional hazard model was used for univariate and multivariate analyses. Two-way ANOVA was used to assess differences between groups. The Student’s t-test was used to determine significant differences in other continuous data. *P*-values less than 0.05 were considered statistically significant.

## Results

### SLCO4A1-AS1 is upregulated in CRC and predicts poor prognosis.

The TCGA CRC dataset including 50 pairs of CRCs and NCTs was analyzed to screen differentially expressed lncRNAs (fold change > 2, P < 0.01) that overlapped with our previous lncRNA expression profile data in CRC [9] (Additional file 4: Fig. S1a). We found that 14 candidate lncRNAs were significantly increased in CRCs compared with paired NCTs (Additional file 3: Table S3). Of these 14 lncRNAs, six (H19, MIR17HG, PVT1, SNHG16, SNHG6, and ZFAS1) have been clearly reported to be up-regulated in CRC. So, we validated the expression of the remaining eight candidate lncRNAs in 12 pairs of CRCs and NCTs using qRT-PCR and revealed that SLCO4A1-AS1 showed the most significant overexpression in CRC compared with other lncRNAs (Additional file 4: Fig. S1b). Therefore, we selected SLCO4A1-AS1 for further research.

**Fig. 1:**
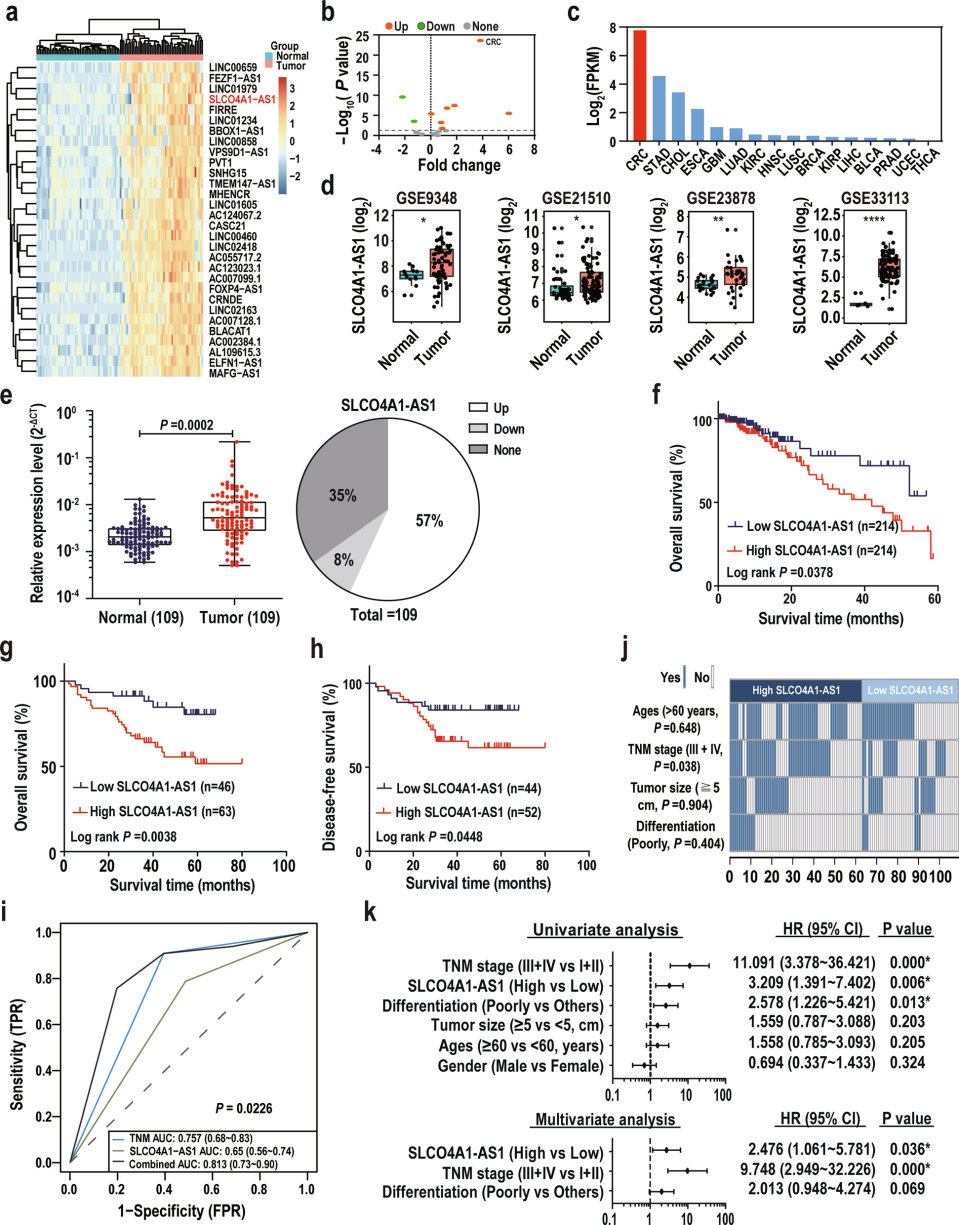
SLCO4A1-AS1 is upregulated in CRC and predicts poor prognosis. **a** Heatmap of the 30 top upregulated lncRNAs from TCGA CRC dataset identified by unsupervised hierarchical clustering. **b** and **c** SLCO4A1-AS1 is overexpressed in multiple cancer types (**b**) and shows high abundance in CRC based on TCGA database (**c**). **d** Validation of SLCO4A1-AS1 overexpression in CRC samples from four independent GEO databases. **e** SLCO4A1-AS1 was upregulated (> two fold) in 57% (62 of 109) of CRC tissues compared with their paired NCTs in CRC cohort 1. Relative expression levels of SLCO4A1-AS1 were quantified by qRT-PCR. **f** Kaplan–Meier survival analysis according to SLCO4A1-AS1 levels in the CRC dataset of TCGA. Overall survival (**g**) and disease-free survival (**h**) analyses according to SLCO4A1-AS1 levels were analyzed using the Kaplan–Meier method. **i** SLCO4A1-AS1-based and TNM-based prediction models were constructed using a ROC method. **j** The associations between different clinical characteristics and SLCO4A1-AS1 expression. Statistical significance was performed using the χ^2^ test. **k** Cox univariate and multivariate regression analyses were performed in CRC cohort 1. *P < 0.05; **P < 0.01

Unsupervised hierarchical clustering with 30 significantly upregulated lncRNAs distinguished the CRCs from corresponding NCTs from TCGA database (Fig. 1a). In addition, pan-cancer analyses based on TCGA datasets revealed the upregulation of SLCO4A1-AS1 in multiple cancer types, with the highest overexpression fold and abundance in CRC (Fig. 1b, c, Additional file 4: Fig. S1c). Moreover, SLCO4A1-AS1 upregulation was also observed in the CRC datasets of GSE9348, GSE21510, GSE23878 and GSE33113 (Fig. 1d). To further validate these results, we examined SLCO4A1-AS1 levels using qRT-PCR in an independent CRC cohort that we collected. Consistent with the results of the online databases, SLCO4A1-AS1 was overexpressed in 57% (62 of 109, fold change > 2) of CRC tissues compared with their corresponding NCTs (P = 0.0002, Fig. 1e).

To evaluate the pathological and clinical value of SLCO4A1-AS1, patients were divided into two groups according to the cutoff value generated by the ROC curve method. Survival analyses revealed that patients with high SLCO4A1-AS1 expression were significantly associated with shorter OS and DFS times in CRC datasets from our hospital and TCGA (Fig. 1f–h). ROC analyses showed that the area under curve (AUC) of the combination of the SLCO4A1-AS1-based and TNM-based prediction models (0.813) was higher than the TNM-based model alone (0.757), suggesting that the combination of SLCO4A1-AS1 and TNM stage is more precise in predicting clinical outcome than TNM stage alone (Fig. 1i). We next assessed and contrasted SLCO4A1-AS1 expression with different clinicopathological features and found that SLCO4A1-AS1 expression positively correlated with TNM stage (Fig. 1j). Furthermore, univariate and multivariate regression analyses elucidated that SLCO4A1-AS1 expression was an independent risk factor for CRC prognosis (Fig. 1k). Collectively, these findings demonstrate that SLCO4A1-AS1 is highly expressed in CRC tissues and predicts poor prognosis.

### Promoter hypomethylation promotes SLCO4A1-AS1 expression in CRC.

**Fig. 2:**
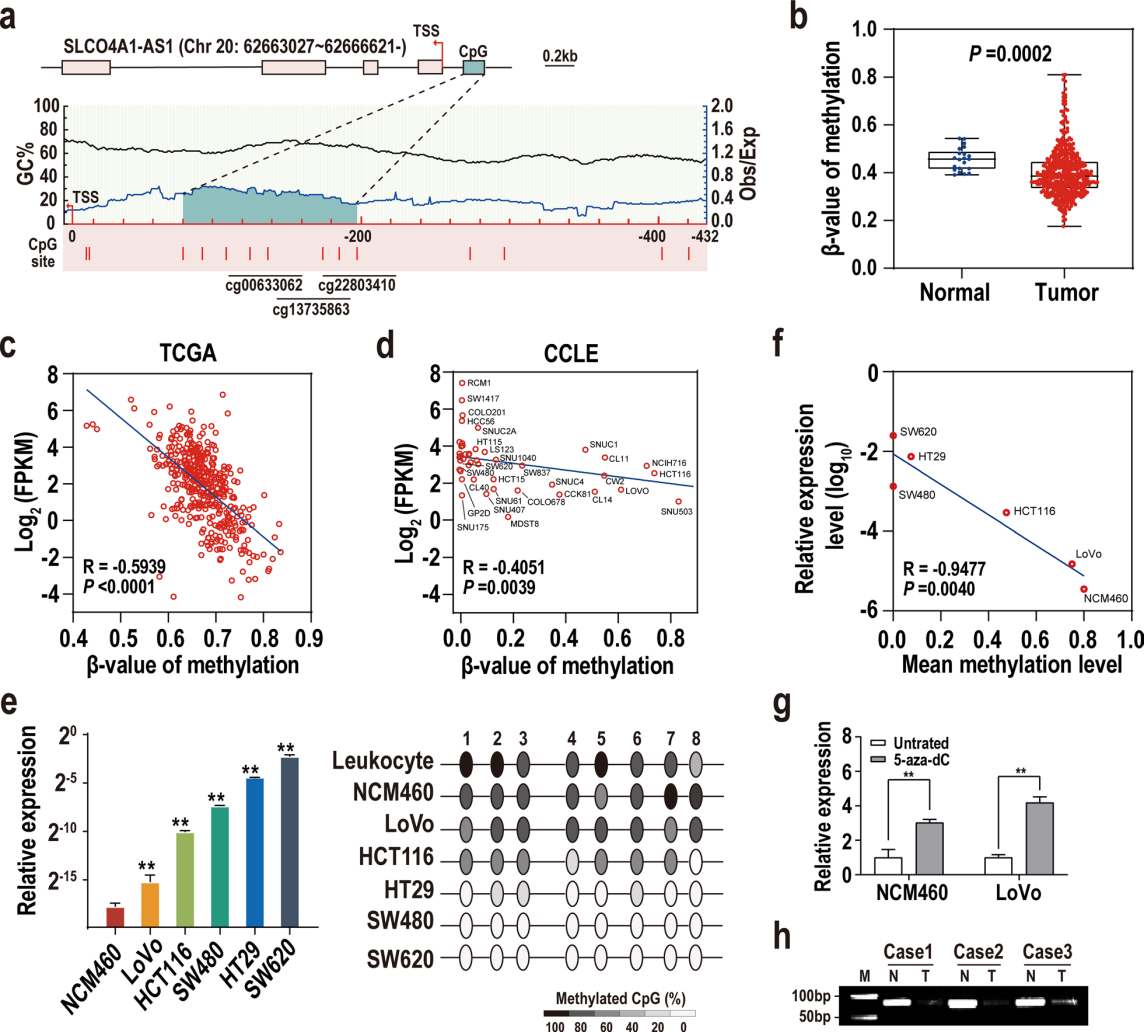
Promoter hypomethylation promotes SLCO4A1-AS1 expression in CRC. **a** Schematic map illustrating a predicted CpG island and its DNA methylation probes in the promoter of SLCO4A1-AS1. TSS, transcription start site. **b** The β-value of methylation of SLCO4A1-AS1 was lower in tumour samples than in normal tissues according to the CRC dataset of TCGA. The β-value of methylation of SLCO4A1-AS1 was linearly related to SLCO4A1-AS1 expression in CRC tissues from TCGA (**c**) and cell lines from CCLE (**d**). **e** Relative expression of SLCO4A1-AS1 in CRC cell lines was measured using qRT-PCR (the left panel). The methylation levels of SLCO4A1-AS1 in CRC cell lines and leukocyte cells were determined by bisulfite sequencing PCR. A total of 5 individual clones were randomly picked for sequencing (the right panel). **f** The mean methylation levels of these CpG sites were negatively associated with the expression levels of SLCO4A1-AS1 in CRC cells. **g** SLCO4A1-AS1 expression in CRC cells treated with DNA methyltransferase inhibitor (5-aza-dC). **h** DNA methylation analyses of SLCO4A1-AS1 in paired CRC tissues and noncancerous tissues using methylation-specific PCR assay. N, adjacent noncancerous tissue; T, tumour tissue; M, DNA marker

Previous studies have shown that promoter hypomethylation is related to the upregulation of oncogene and subsequent tumourigenesis [26]. We next sought to investigate the effect of promoter methylation status on the expression of SLCO4A1-AS1. Interestingly, we identified one CpG island located in the promoter of SLCO4A1-AS1 using MathPrimer, which was successfully detected by three DNA methylation probes (cg00633062, cg22803410, and cg13735863) (Fig. 2a). According to TCGA dataset, the methylation levels of SLCO4A1-AS1 in CRC tissues were markedly lower than those in NCTs and were negatively correlated with the expression levels of SLCO4A1-AS1 in CRC tissues from both TCGA and CCLE data sets (Fig. 2b–d, Additional file 5: Table S4). The methylation status of the CpG island was examined by BSP in CRC cell lines and two types of normal control cells (Leukocyte and NCM460). The results showed that the CpG sites in this CpG island were rarely methylated in SW620, SW480 or HT29 cells, with average methylation rates of 0, 0 and 7.5%, respectively (Fig. 2e). In contrast, the mean methylation ratios of these CpG sites were especially high in normal cells (80% for NCM460 and 82.5% for human leukocytes, Fig. 2e). What is more, the negative association between the methylation status and expression of SLCO4A1-AS1 was also confirmed in these CRC cell lines (Fig. 2f). Additionally, NCM460 and LoVo cells with low expression of SLCO4A1-AS1 were treated with the DNA methyltransferase inhibitor 5-aza-dC, and the expression of SLCO4A1-AS1 in the treated cells was significantly restored (Fig. 2g). The promoter methylation status of SLCO4A1-AS1 was also investigated in several paired tumour tissues using an MSP assay. The results showed that the relative methylation levels of SLCO4A1-AS1 promoter were significantly decreased in CRCs compared with NCTs (Fig. 2h). Together, these data demonstrate that promoter hypomethylation is an important mechanism mediating the upregulation of SLCO4A1-AS1 in CRC.

### SLCO4A1-AS1 induces CRC cell proliferation in vitro and in vivo

**Fig. 3:**
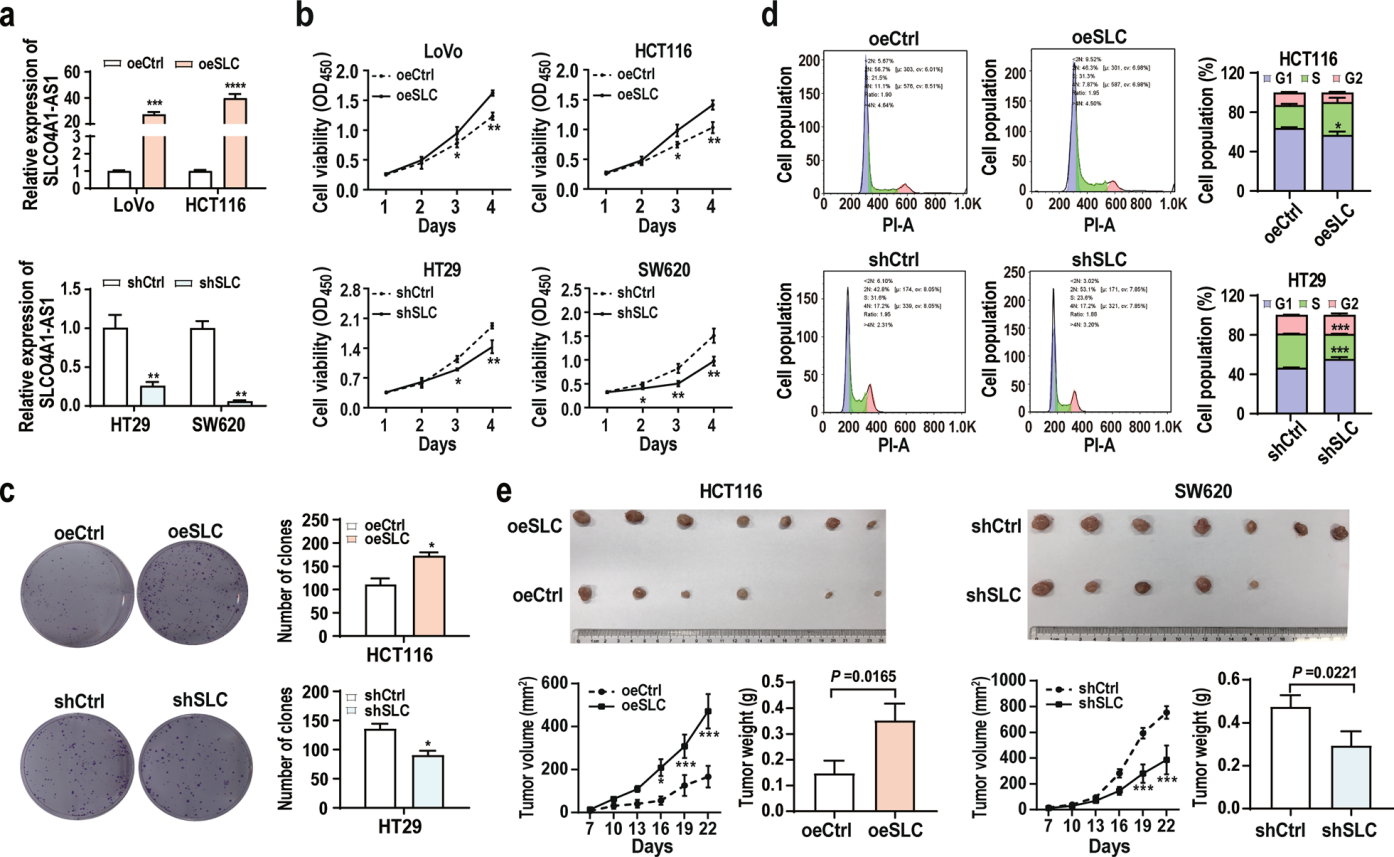
SLCO4A1-AS1 promotes CRC proliferation in vitro and in vivo. **a** Validation of SLCO4A1-AS1 expression in CRC cells transfected with SLCO4A1-AS1 overexpression (oeSLC) or shRNA (shSLC) plasmids. **b** The effects of SLCO4A1-AS1 on CRC cell proliferation were assessed by CCK-8 assays. **c** Effects of SLCO4A1-AS1 on the colony formation abilities of CRC cells. **d** The effects of SLCO4A1-AS1 on the cell cycle in CRC cells were evaluated by FACS. **e** The effect of SLCO4A1-AS1 on CRC tumour formation in a nude mouse xenograft model. Representative images of tumours from the SLCO4A1-AS1 (shSLC and oeSLC) and control groups (n = 7 for each group). *P < 0.05; **P < 0.01; ***P < 0.001

To explore the effects of SLCO4A1-AS1 on cell functions, we overexpressed SLCO4A1-AS1 in HCT116 and LoVo cells expressing relatively low levels of endogenous SLCO4A1-AS1 and knocked down SLCO4A1-AS1 expression in HT29 and SW620 cells with relatively high levels of endogenous SLCO4A1-AS1 (Figs. 2e and 3a). The results showed that overexpression of SLCO4A1-AS1 dramatically promoted cell proliferation and colony formation of HCT116 and LoVo cells, whereas knockdown of SLCO4A1-AS1 significantly impaired cell proliferation and colony formation of HT29 and SW620 cells (Fig. 3b, c). Moreover, ectopic SLCO4A1-AS1 expression promoted G1-S cell cycle progression, whereas silencing SLCO4A1-AS1 expression decreased the number of cells in the S phase compared with their control cells (Fig. 3d). Furthermore, SLCO4A1-AS1 overexpression promoted CRC tumourigenicity, whereas silencing SLCO4A1-AS1 inhibited CRC tumour growth in nude mice (Fig. 3e). Collectively, these data demonstrate that SLCO4A1-AS1 promotes CRC cell proliferation and tumourigenicity.

### SLCO4A1-AS1 interacts with Hsp90 and Cdk2 in CRC

**Fig. 4:**
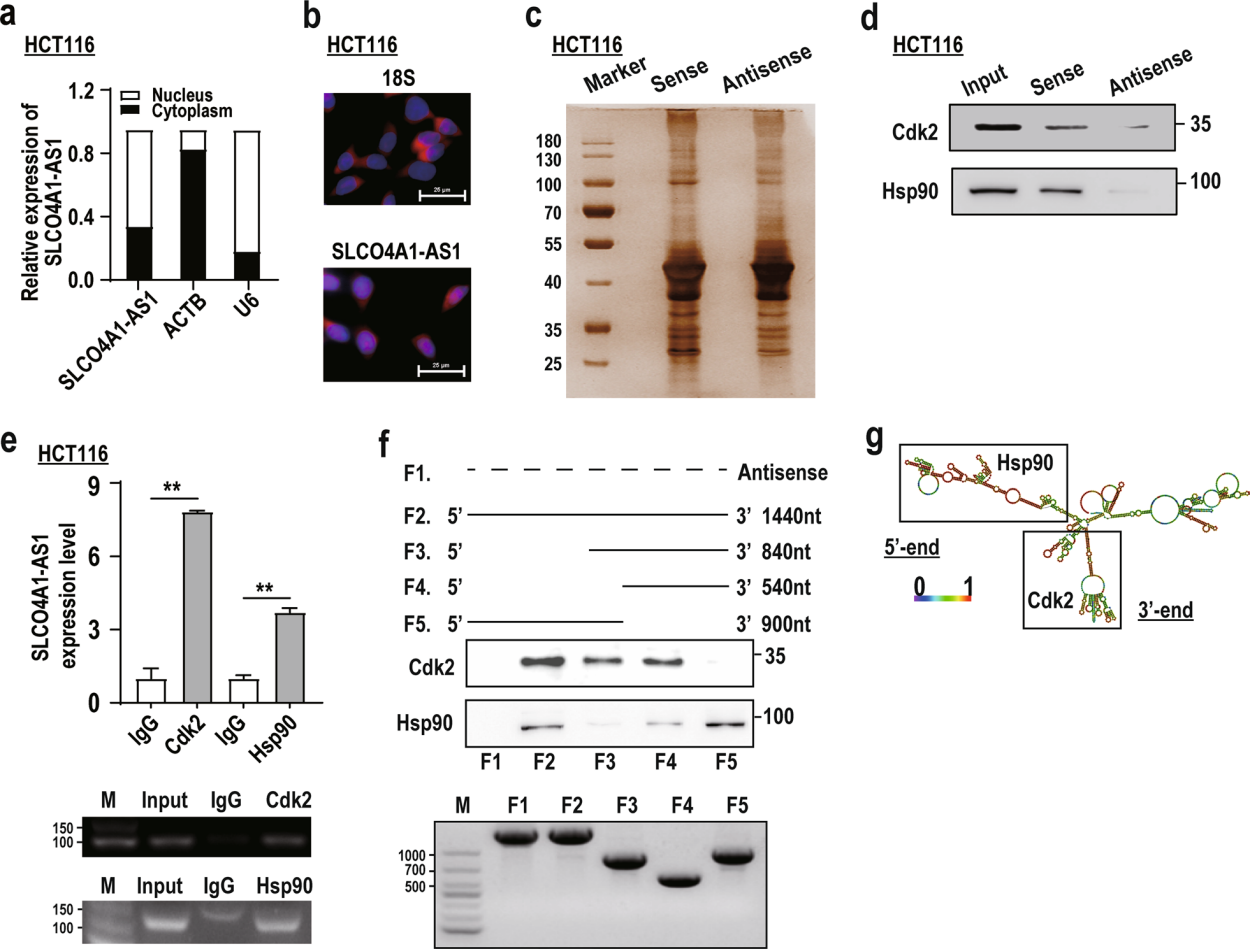
SLCO4A1-AS1 interacts with Hsp90 and Cdk2 in CRC. **a** and **b** Subcellular localization of SLCO4A1-AS1 was determined by qRT-PCR (**a**) and FISH (**b**) in HCT116 cells. **c** Proteins retrieved from the SLCO4A1-AS1 pull-down assay were analyzed by SDS-PAGE in HCT116 cells. **d** Western blotting analyses of Hsp90 and Cdk2 using the proteins retrieved from the SLCO4A1-AS1 pull-down assay in HCT116 cells. **e** RIP assays using an anti-Cdk2 or anti-Hsp90 antibody showed that both Cdk2 and Hsp90 interact with SLCO4A1-AS1 in HCT116 cells. The qRT-PCR results of RIP assays are shown in the upper panel, and the agarose electrophoresis results of the PCR products are shown in the lower panel. **f** Western blotting of Cdk2 and Hsp90 in protein samples pulled down by the full-length (F2:1–1440 bp) or truncated SLCO4A1-AS1 (F3: 600–1440 bp, F4: 900–1440 bp, F5: 1–900 bp) (the upper panel), and truncation mutants of SLCO4A1-AS1 shown by agarose electrophoresis (the lower panel). **g** Graphic illustration of the secondary structure of SLCO4A1-AS1 predicted by ViennaRNA (http://rna.tbi.univie.ac.at/) and its corresponding regions accounting for the binding with Cdk2 and Hsp90. *P < 0.05; **P < 0.01

To further identify targets directly interacting with SLCO4A1-AS1, we firstly investigated the subcellular localization of SLCO4A1-AS1 using qRT-PCR and FISH. The results showed that SLCO4A1-AS1 was localized in both the nucleus and cytoplasm in CRC cells and tissues (Fig. 4a, b, and Additional file 6: Fig. S2a and b). We next pulled down SLCO4A1-AS1 binding proteins in HCT116 and SW620 cells using RNA pull-down assays. The retrieved proteins were separated using SDS-PAGE gels (Fig. 4c and Additional file 6: Fig. S2c), and several differential gel bands from HCT116 cells were chosen for mass spectrum analyses, and the potential interacting proteins were ranked by largest based on a confidence score in mass spectrometric assays (Additional file 7: Table S5). Among these proteins, Hsp90 (HSP90AB1) could function as a molecular chaperone to control protein kinase ubiquitination degradation, regulating cell survival and carcinogenesis [27–29], whereas Cdk2, a client protein of Hsp90, is widely known as a protein kinase involved in cell cycle regulation and tumourigenesis [30], which is consistent with the aforementioned phenotypic results of SLCO4A1-AS1. Therefore, we selected Hsp90 and Cdk2 for further validations. Western blotting analyzes confirmed that both Hsp90 and Cdk2 were bound to SLCO4A1-AS1 using the retrieved proteins in the RNA pull-down assay (Fig. 4d and Additional file 6: Fig. S2d). These interactions were further confirmed using RIP assays (Fig. 4e).

To identify the Hsp90- or Cdk2-interacting region in SLCO4A1-AS1, we constructed three deletion mutants of SLCO4A1-AS1 based on the predicted secondary structure of SLCO4A1-AS1 using ViennaRNA (http://rna.tbi.univie.ac.at/) (Fig. 4f, g). The results of pull-down assays showed that the 5’-terminus (1–600 bp) of SLCO4A1-AS1 mediated its interaction with Hsp90, and the 3’-terminus (900–1440 bp) was essential for the binding of SLCO4A1-AS1 to Cdk2 (Fig. 4f, g). Taken together, these data demonstrate that SLCO4A1-AS1 physically associates with both Hsp90 and Cdk2 proteins.

### SLCO4A1-AS1 acts as a scaffold in the Hsp90/Cdk2 complex to regulate Cdk2 stability

**Fig. 5:**
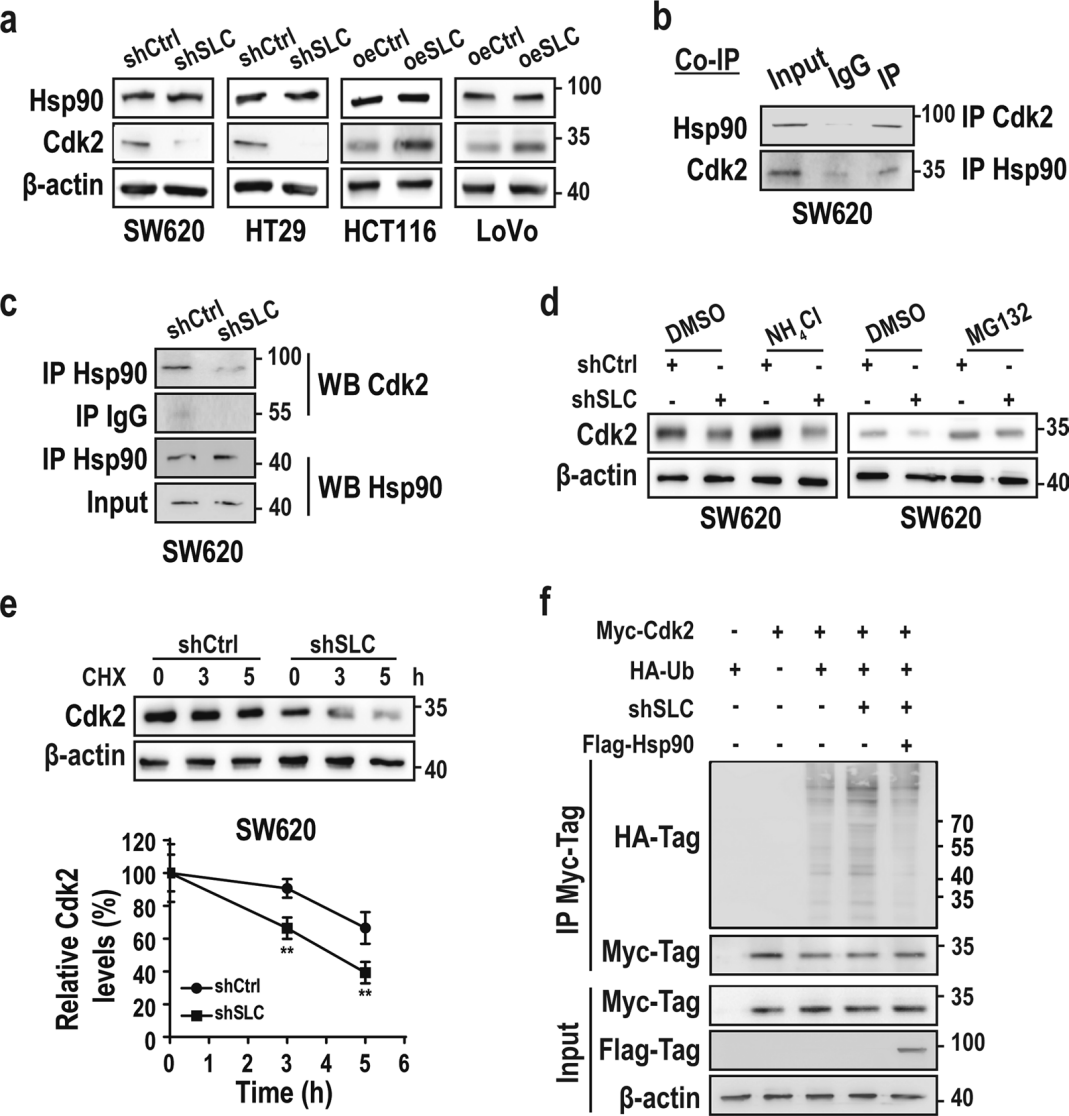
SLCO4A1-AS1 acts as a scaffold to promote the association between Cdk2 and Hsp90. **a** Protein expression of Cdk2 and Hsp90 in the SLCO4A1-AS1 overexpressing or SLCO4A1-AS1-depleted CRC cells. **b** Co-immunoprecipitation (Co-IP) assays were performed to detect the interaction between Hsp90 and Cdk2. **c** SLCO4A1-AS1 knockdown impairs the interaction between Hsp90 and Cdk2 in CRC cells. **d** Immunoblot analysis of Cdk2 in SW620 cells transfected with shCtrl and shSLC plasmids. Cells were treated with NH4Cl (25 mM) or MG132 (20 μM) for 3 h before harvest. **e** SLCO4A1-AS1 knockdown decreased the protein stability of Cdk2. Cells were treated with CHX (50 μg/ml) as indicated before harvest. **f** Hsp90 blocked SCLO4A1-AS1 knockdown-induced ubiquitination modification of Cdk2. 293 T cells were co-transfected with different plasmids as indicated, and their lysates were subjected to protein ubiquitination assays using Myc-Tag beads. *P < 0.05; **P < 0.01

We next determined whether SLCO4A1-AS1 regulates Hsp90 and/or Cdk2 activities and found that overexpression or knockdown of SLCO4A1-AS1 did not affect the protein levels of Hsp90 in CRC cells (Fig. 5a). Nevertheless, overexpression of SLCO4A1-AS1 increased, while SLCO4A1-AS1 knockdown decreased Cdk2 protein expression (Fig. 5a). As a client of the Hsp90, Cdk2 protein levels are markedly increased by Hsp90 [30]. Therefore, we hypothesized that SLCO4A1-AS1 may act as a scaffold to regulate the association between Hsp90 and Cdk2, resulting in enhanced Cdk2 activity. To test this hypothesis, we performed co-IP assays and confirmed the association between Hsp90 and Cdk2 (Fig. 5b), which was significantly impaired by SLCO4A1-AS1 knockdown (Fig. 5c). In line with these results, we revealed that the proteasome inhibitor MG132 efficiently restored Cdk2 expression in SLCO4A1-AS1-depleted CRC cells, whereas the lysosome inhibitor NH4Cl was unable to block SLCO4A1-AS1 knockdown-induced degradation of Cdk2 (Fig. 5d). Furthermore, the protein synthesis inhibitor cycloheximide (CHX) was used to evaluate the effect of SLCO4A1-AS1 on the stability of Cdk2. We observed that silencing SLCO4A1-AS1 expression in CHX-treated SW620 cells shortened the half-life of Cdk2 compared to that of the control group (Fig. 5e). Further ubiquitination analyses showed that SLCO4A1-AS1 knockdown significantly promoted Cdk2 ubiquitination, which was blocked by ectopic expression of Hsp90 (Fig. 5f). Together, these data suggest that SLCO4A1-AS1 functions as a scaffold to strengthen the interaction between Hsp90 and Cdk2, inhibiting the ubiquitination and degradation of Cdk2 in CRC cells.

### SLCO4A1-AS1 mediates multiple cancer-related signalling pathways

**Fig. 6:**
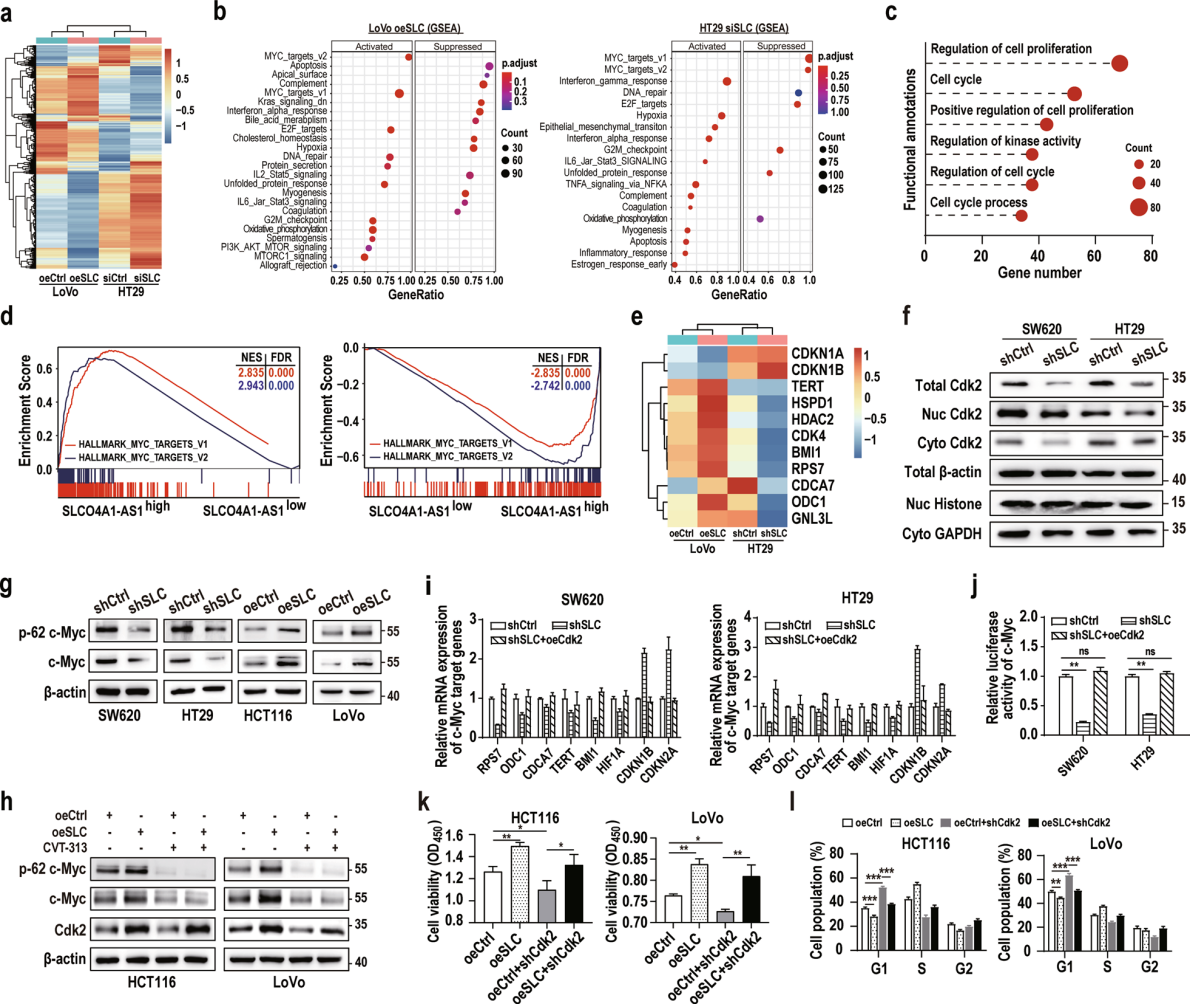
SLCO4A1-AS1 activates c-Myc signalling in a Cdk2-dependent manner in CRC. **a** Gene expression profiles of CRC cells transfected with SLCO4A1-AS1 plasmids (oeSLC) or siSLCO4A1-AS1 (siSLC). **b** GSEA focused on a set of signalling pathways after SLCO4A1-AS1 overexpression and SLCO4A1-AS1 silencing, summarized based on the enrichment score. **c** Functional annotation clustering of common DEGs regulated by SLCO4A1-AS1 in two independent RNA sequencing assays is shown. The x-axis shows the ratio of the number of genes in a given category of functional annotations divided by the total number of DEGs. The y axis shows categories of functional annotations. **d** GSEA showed that high SLCO4A1-AS1 expression was positively related to the activation of c-Myc signalling. **e** Expression heatmap of the target genes of c-Myc signalling shown in two independent RNA sequencing assays. **f** Western blotting showing that shRNA-induced SLCO4A1-AS1 knockdown decreased the protein levels of Cdk2 in SW620 and HT29 cells. Histone (Histone H3.1), nuclear marker; GAPDH, cytoplasmic marker; Nuc, Nucleus; Cyto, Cytoplasm. **g** The c-Myc proteins were analyzed by western blotting using phospho (Ser62)- and pan-Myc antibodies in CRC cells transfected with control or SLCO4A1-AS1 (shSLC or oeSLC) plasmids. **h** Western blotting of proteins from the control or shSLCO4A1-AS1 group was performed in HCT116 and LoVo cells with or without CVT-313 treatment. **i** The mRNA levels of downstream target genes of the c-Myc pathway were measured by qRT-PCR in CRC cells transfected with control and SLCO4A1-AS1-depleted plasmids or cotransfected with Cdk2 overexpression plasmids. **j** Luciferase assays were performed to assess the effects of SLCO4A1-AS1 and Cdk2 on the c-Myc pathway. **k** Cell viability was measured by CCK-8 assays in CRC cells. **l** FACS analyses of the effects of SLCO4A1-AS1 on the cell cycle in HCT116 and LoVo cells. *P < 0.05; **P < 0.01; ***P < 0.001

To gain further insight into the SLCO4A1-AS1-regulated signalling pathways involved in CRC pathogenesis, we measured unbiased transcriptome profiling in SLCO4A1-AS-overexpressing LoVo cells or SLCO4A1-AS1-depleted HT29 cells using RNA-seq. Compared to the negative control, genes with opposite expression trends in the two independent RNA sequencing datasets were selected and subjected to hierarchical clustering and pathway enrichment analyses (Fig. 6a and Additional file 8: Table S6). GSEA results revealed that these genes are involved in several signalling pathways, including the c-Myc targets pathway (activated), apoptosis (suppressed), DNA repair (activated), G2M checkpoint (activated), and oxidative phosphorylation (activated), which are closely related to cell survival and tumourigenesis (Fig. 6b). In addition, DEGs ((|logFC|≧0.5) were further selected for GO analyses, and the results showed that these DEGs were enriched in several biological processes involving cell proliferation, cell cycle, and regulation of kinase activity (Fig. 6c). Notably, GSEA functional annotation analyses showed that several MYC-related pathways, such as the MYC_targets_V1 pathway and the MYC_targets_V2 pathway, were among the top-scoring processes, suggesting that SLCO4A1-AS1 might be involved in the transcriptional activation of c-Myc target genes (Fig. 6d, e). In addition, both total and nuclear Cdk2 levels were significantly decreased in SLCO4A1-AS1-silenced CRC cells (Fig. 6f). Overall, these data suggest that SLCO4A1-AS1 regulates multiple cancer-related pathways, especially c-Myc-related pathways.

### SLCO4A1-AS1 activates c-Myc signalling in a Cdk2-dependent manner in CRC

Cdk2 could promote c-Myc-mediated transcription by phosphorylating c-Myc protein at Ser62 and increasing its stability [31, 32]. Therefore, we hypothesized that SLCO4A1-AS1 exerts its functions by promoting Cdk2-mediated c-Myc Ser62 phosphorylation (p-62 c-Myc). As expected, SLCO4A1-AS1 knockdown reduced, whereas ectopic SLCO4A1-AS1 expression increased the levels of both c-Myc and p-62 c-Myc (Fig. 6g). Moreover, CVT-313 (a selective inhibitor of Cdk2) treatment blocked the SLCO4A1-AS1-induced elevation of c-Myc and p-62 c-Myc (Fig. 6h) in CRC cells. As expected, we observed that ectopic expression of Cdk2 rescued the expression of c-Myc target genes in SLCO4A1-AS-depleted CRC cells (Fig. 6i). In addition, we constructed a c-Myc reporter plasmid and evaluated the activation status of the c-Myc pathway in CRC cells using luciferase reporter assays. The results showed that c-Myc signalling was significantly inhibited in SLCO4A1-AS1-silenced CRC cells, which was rescued by forced overexpression of Cdk2 (Fig. 6j). Consistent with these results, SLCO4A1-AS1-induced CRC proliferation and cell cycle progression were significantly inhibited by Cdk2 knockdown (Fig. 6k, l). Collectively, these data demonstrate that SLCO4A1-AS1 exerts tumour-promoting functions in CRC by activating the Cdk2/c-Myc axis.

### Cdk2 and p-62 c-Myc expression correlates with SLCO4A1-AS1 and prognosis in CRC

**Fig. 7:**
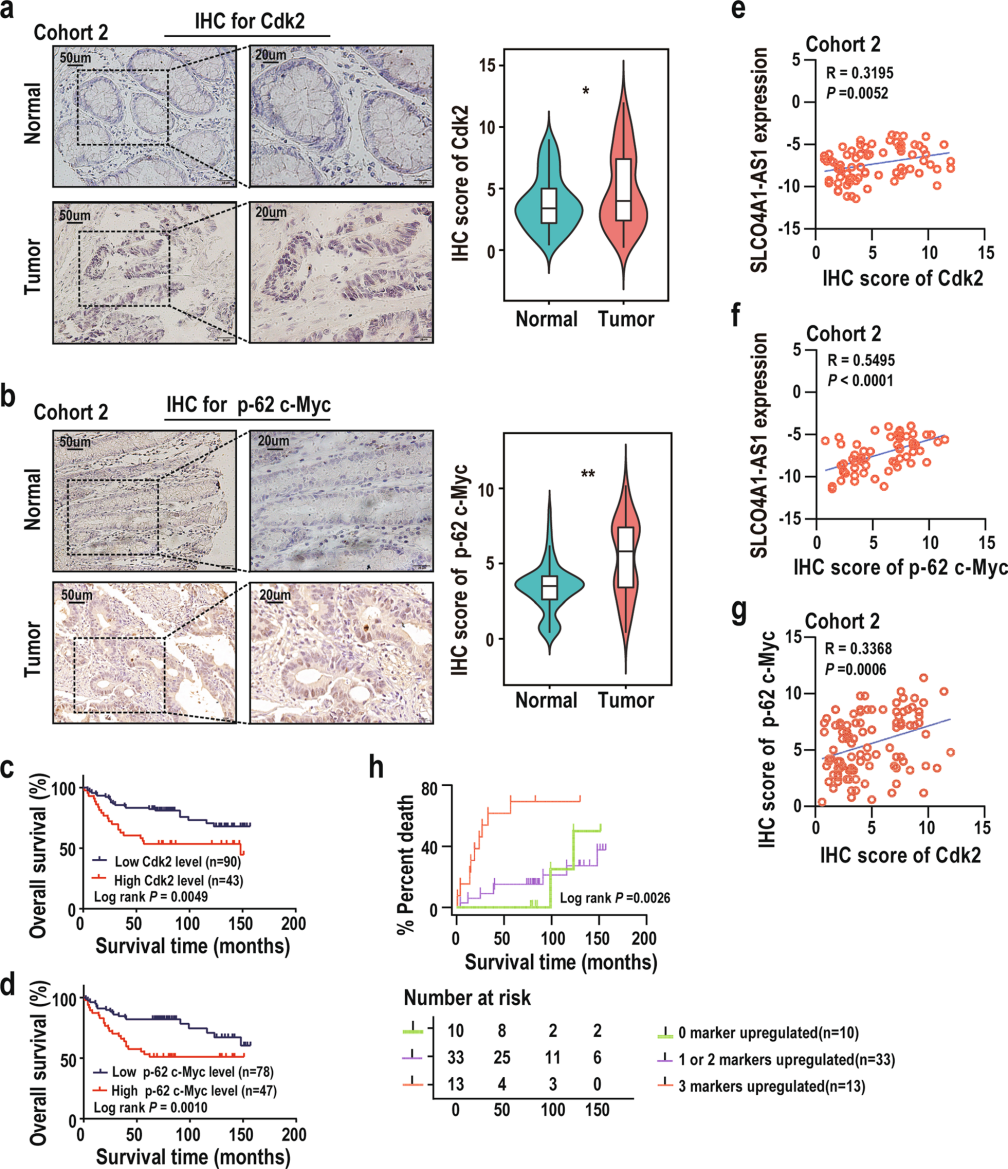
Levels of SLCO4A1-AS1, Cdk2, and p-62 c-Myc are correlated and predict CRC patient outcome. **a** and **b** Representative images and statistical analysis of Cdk2 (a, paired n = 97) and p-62 c-Myc (b, paired n = 98) expression by IHC in tumour tissues and paired normal tissues in CRC cohort 2. Overall survival analyses according to Cdk2 (**c**, n = 133) and p-62 c-Myc (**d**, n = 125) expression were analyzed by Kaplan–Meier method in a CRC cohort 2. **e** Correlation analysis of SLCO4A1-AS1 abundance and Cdk2 expression in CRC cohort 2 (n = 75, Pearson correlation test). **f** Correlation analysis of SLCO4A1-AS1 abundance and p-62 c-Myc expression in CRC cohort 2 (n = 62, Pearson correlation test). **g** Correlation analysis of Cdk2 and p-62 c-Myc expression in CRC cohort 2 (n = 100, Pearson correlation test). **h** Kaplan–Meier analysis of overall survival in CRC patients based on the number of upregulated markers (SLCO4A1-AS1, Cdk2 and p-62 c-Myc; n = 56, Log-rank test). *P < 0.05; **P < 0.01

To investigate the correlations and prognostic value of SLCO4A1-AS1, Cdk2 and p-62 c-Myc in patients with CRC, we simultaneously measured their levels in CRC tissues. We found that the protein levels of Cdk2 and p-62 c-Myc were significantly elevated in CRC tissues compared to those in NCTs and predicted poor prognosis (Fig. 7a–d), and Correlation analyses indicated that the expression of SLCO4A1-AS1, Cdk2, and p-62 c-Myc was significantly correlated with each other (Fig. 7e–g). Furthermore, using SLCO4A1-AS1, Cdk2, and p-62 c-Myc as three risk factors, we tracked the cumulative mortality rate in CRC patients. The results showed that the shortest OS time was observed in patients with high levels of these three markers (Fig. 7h). Taken together, these clinical analyses confirm the regulatory axis of the SLCO4A1-AS1/Cdk2/ p-62 c-Myc in CRC.

## Discussion

**Fig. 8:**
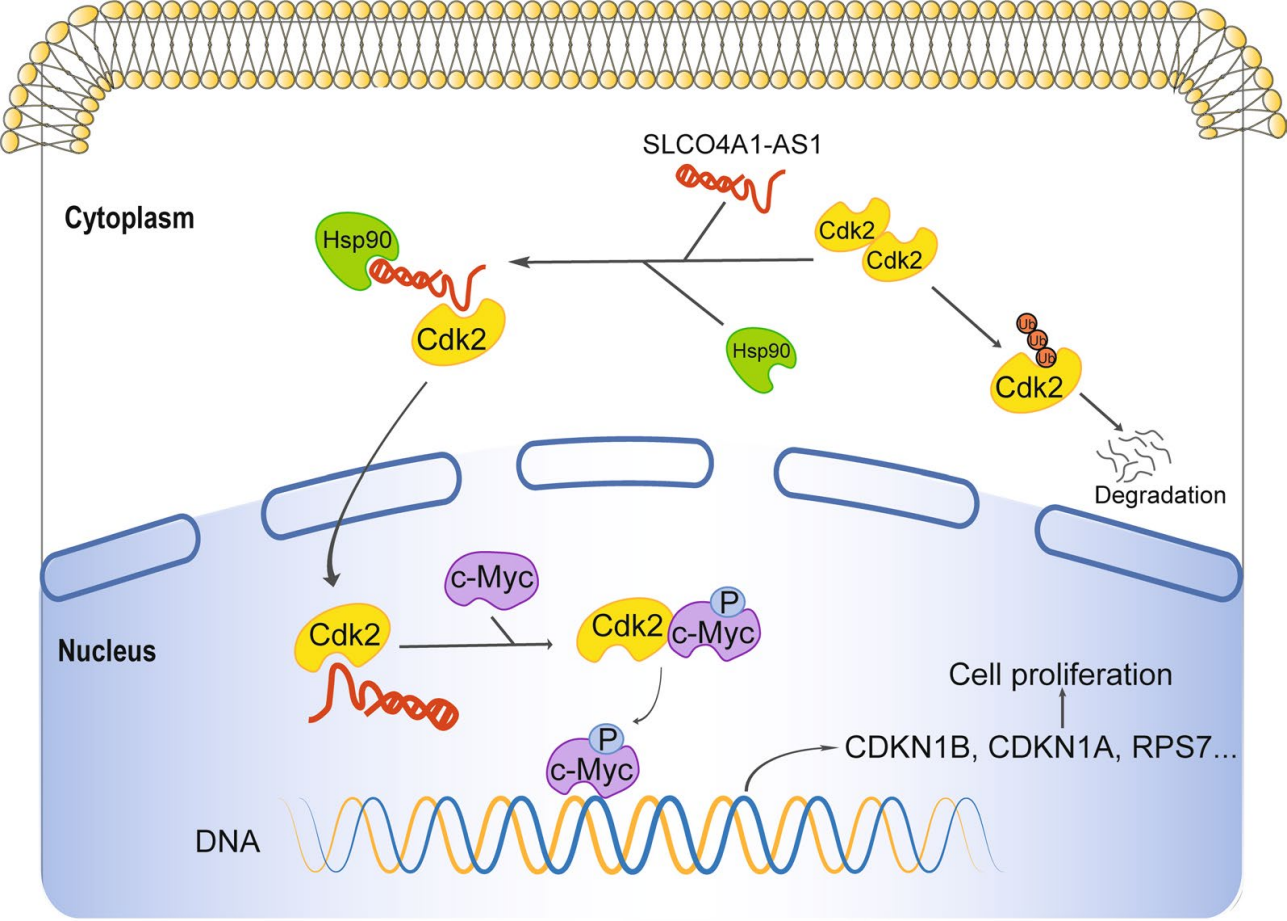
Schematic representation for the mechanism of the SCLCO4A1-AS1/Hsp90/Cdk2/c-Myc axis in CRC

Accumulating studies have shown that dysregulated lncRNAs play important roles by participating in multiple carcinogenic pathways. SLCO4A1-AS1 is a newly identified oncogenic lncRNA in multiple cancer types [16, 18–21, 33]. In this study, we confirmed that the aberrant overexpression of SLCO4A1-AS1 is associated with poor prognosis in multiple CRC cohorts and revealed that promoter hypomethylation is a key mechanism mediating the upregulation of SLCO4A1-AS1 in CRC. Using a combination of genomic, biochemical, and cell biology analyses, we demonstrated that SLCO4A1-AS1 promotes colorectal tumourigenesis by strengthening the binding of Hsp90 to Cdk2, resulting in increased Cdk2 stability and subsequent activation of the c-Myc signalling pathway (Fig. 8).

Li and colleagues first reported that SLCO4A1-AS1 is overexpressed in CRC [34]. Other groups further confirmed the aberrant expression of SLCO4A1-AS1 in CRC, which was linked to poor survival [20, 21]. Elevated expression of SLCO4A1-AS1 was also observed in bladder cancer [33], lung cancer [16, 18], and laryngeal squamous cell carcinoma [35]. In this study, we also observed that SLCO4A1-AS1 is upregulated in multiple cancer types, especially digestive system cancers. Clinical sample validation in multiple CRC cohorts further confirmed the overexpression of SLCO4A1-AS1 in CRC and increased SLCO4A1-AS1 levels were associated with poor survival. In addition, we observed that the combination of SLCO4A1-AS1 and TNM stage was more precise for predicting clinical outcome than TNM stage alone.

Little is known about the mechanism mediating SLCO4A1-AS1 overexpression in human cancers. Yu et al. [21] reported that copy number amplification of SLCO4A1-AS1 was identified in approximately 9% of CRC tissues based on TCGA database. Promoter CpG methylation is a key mechanism regulating gene expression, including lncRNAs [26, 36]. In this study, we demonstrated that a CpG island in the promoter region of SLCO4A1-AS1 is hypermethylated in normal cells but hypomethylated in CRC, and its methylation levels were negatively correlated with SLCO4A1-AS1 expression in two independent CRC cohorts and CRC cell lines. These data reveal, for the first time, that promoter hypomethylation is an important mechanism mediating the upregulation of SLCO4A1-AS1 in CRC.

Several studies have reported the regulatory roles of SLCO4A1-AS1 in tumour cell proliferation, apoptosis, autophagy and metastasis in several cancer types, including bladder cancer, CRC, lung cancer, and laryngeal squamous cell carcinoma [16, 18–21, 33]. In this study, we further showed that SLCO4A1-AS1 promotes CRC proliferation and cell cycle progression in vitro, and tumourigenesis in vivo. To date, SLCO4A1-AS1-related mechanistic investigations have focused on miRNAs, and some papers have revealed that SLCO4A1-AS1 functions as a competing endogenous RNA (ceRNA) to inhibit the activities of several miRNAs, including miR-223-3p [18], miR-150-3p [17], miR-4701-5p [16], miR-335-5p [33], miR-876-3p [37], and miR-508-3p [19]. Notably, in addition to miR-223-3p, the abundance of other miRNAs is very low in CRC tissues (data not shown), suggesting that these miRNAs are not key molecules mediating the tumour-promoting functions of SLCO4A1-AS1 under physiological and pathological conditions. In addition, Wu et al. reported that SLCO4A1-AS1 competitively binds to miR-150-3p to elevate SLCO4A1 expression. Interestingly, although we also observed a positive correlation between SLCO4A1-AS1 expression and SLCO4A1 mRNA levels in CRC tissues, force expression or knockdown of SLCO4A1-AS1 in CRC cells exhibited weak or no significant effect on the expression of SLCO4A1 (Additional file 9: Fig. S3a, b). Because the endogenous expression of SLCO4A1 is more abundant than that of SLCO4A1-AS1 in CRC cells (data not shown), we speculated that this effect of SLCO4A1-AS1 on SLCO4A1 is not a primary mechanism mediating the tumour-promoting functions of SLCO4A1 in CRC.

Several studies have reported that SLCO4A1-AS1 promotes tumour growth and metastasis by potentially influencing the β-catenin/Wnt and EGFR/MAPK signal pathways [20, 21, 35]. However, how SLCO4A1-AS1 regulates these pathways remains to be elucidated, and little is known about the direct downstream targets of SLCO4A1-AS1. To the best of our knowledge, the only known SLCO4A1-AS1-associated protein is β-catenin, and Yu et al. revealed that SLCO4A1-AS1 increases the stability of β-catenin by inhibiting its phosphorylation by GSKβ, resulting in CRC progression [21].

To investigate the underlying mechanisms by which SLCO4A1-AS1 regulate CRC growth and cell cycle progression, we analyzed signalling pathways potentially regulated by SLCO4A1-AS1 using RNA-seq. We identified that several signalling pathways, including the c-Myc targets, cell cycle, apoptosis, and DNA repair, were significantly altered in CRC cells in response to SLCO4A1-AS1 overexpression or knockdown. We further identified two SLCO4A1-AS1-associated proteins, Cdk2 and Hsp90, and elucidated the exact interacting regions mediating their associations. Cdk2 involves numerous oncogenic pathways, especially the cell cycle and DNA replication signallings [38]. The Hsp90 chaperone also plays a key role in the survival of cancer cells. Many clients of Hsp90, including telomerase, p53, protein kinases, HIF1α, MDM2 and STAT3, are important factors regulating tumourigenesis [27]. Interestingly, Cdk2 has been reported to be a client of Hsp90, and Hsp90 inhibitor treatment significantly reduces levels of Cdk2 in cancer cells [30]. By a series of assays, we revealed that SLCO4A1-AS1 inhibits Cdk2 ubiquitination/degradation by strengthening the association between Hsp90 and Cdk2 as a molecular scaffold. Both Cdk2 and Hsp90 are candidates for cancer therapy, and some clinical trials are currently in progress using Cdk2 or Hsp90 inhibitors. However, it is challenging to develop selective Cdk2 inhibitors [38], and the therapeutic effects of most Hsp90 inhibitors are also unsatisfactory. Pashtan et al. [39] reported that the simultaneous inhibition of Hsp90 and tyrosine kinase that reduces oncogene switching and drug resistance in breast cancer cells, which highlights targeting SLCO4A1-AS1 as an alternative strategy to inhibit both Cdk2 and Hsp90.

Dysregulation of the proto-oncogene MYC family was observed in more than 50% of human cancers and is frequently associated with poor clinical outcomes. Myc plays a pivotal role in almost every aspect of oncogenic processes, including proliferation, cell cycle progression, apoptosis, metabolism, and cancer immune response. For example, in CRC, frequent defects of the Wnt-APC pathway result in enhanced transcriptional activation of MYC. As a key member of the Myc family, c-Myc also contributes to extensive dysregulated cell growth and oncogenesis, especially in CRC development [40]. However, developing drugs directly targeting Myc is challenging due to its “undruggable” protein structure. Cdk2 phosphorylates c-Myc at Ser62 to suppress its ubiquitination modification/degradation, resulting in enhanced stability of c-Myc [31]. Targeting Cdk2 shows promising prospects for human cancers, especially in c-Myc-overexpressing cancers [38]. Here, we revealed that SLCO4A1-AS1 exerts tumour-promoting functions by regulating the Cdk2-c-Myc axis in CRC, suggesting that targeting SLCO4A1-AS1 appears to be an alternative strategy for inhibiting the Cdk2-c-Myc axis in cancer therapy.

## Conclusions

In conclusion, we confirmed the aberrant upregulation of SLCO4A1-AS1 in CRC and uncovered that promoter hypomethylation at least partly explains the deregulated expression of SLCO4A1-AS1 in CRC. SLCO4A1-AS1 promotes colorectal tumourigenesis by enhancing the interaction between Cdk2 and Hsp90, resulting in increased Cdk2 levels and consequently activated c-Myc signalling in CRC cells. Our data suggest that targeting SLCO4A1-AS1 may represent a promising strategy for CRC treatment.

## Supplementary Information

**Additional file 1: Table S1.** Clinicopathologic features of CRC patients.

**Additional file 2: Table S2.** List of primers.

**Additional file 3: Table S3.** Identificantion of fourteen lncRNAs candidates upregulation from 5 and 50 pairs CRC and adjacent tissues

**Additional file 4: Fig. S1.** SLCO4A1-AS1 is upregulated in CRC and predicts poor prognosis. **a** The flow chart for selected candidate lncRNAs overexpressed in CRC compared to adjacent tissues from TCGA and our previous microarray datasets. **b** The expression of eight candidate lncRNAs was quantified in 12 paired CRC tissues and their adjacent tissues. **c** SLCO4A1-AS1 was overexpressed in multiple cancer types from TCGA databases. **d** Relative expression levels of SLCO4A1-AS1 were quantified by qRT-PCR in CRC cohort 2. **e** and **f** Overall survival (e) and Disease-free survival (f) according to SLCO4A1-AS1 levels were analyzed by Kaplan–Meier method in CRC cohort 2. *P < 0.05; **P < 0.01; ***P < 0.001.

**Additional file 5: Table S4.** The related original data of Fig. 2d.

**Additional file 6: Fig. S2.** Subcellular localization and binding proteins of SLCO4A1-AS1 in CRC. **a** Subcellular localization of SLCO4A1-AS1 was determined by qRT-PCR in HT29 and SW620 cells **b** Subcellular localization of SLCO4A1-AS1 was determined by FISH in CRC tissues **c** Proteins retrieved from the SLCO4A1-AS1 pull-down assay were analyzed by SDS-PAGE in SW620 cells. **d** Western blotting analyses of Hsp90 and Cdk2 using the proteins retrieved from the SLCO4A1-AS1 pull-down assay in SW620 cells. *P < 0.05; **P < 0.01; ***P < 0.001.

**Additional file 7: Table S5.** The top proteins specific to SLCO4A1-AS1 identified in sense RNA group bands at approximately 100 kD and 35 kD by Mass Spectrometry analysis.

**Additional file 8: Table S6.** A total of 754 regulated DEGs (|logFC|≧0.5) selected after both overexpression and knockdown of SLCO4A1-AS1 in two independent RNA sequencing data sets.

**Additional file 9: Fig. S3.** The relationship between SLCO4A1-AS1 and SLCO4A1 in CRC. **a** Correlation analysis of SLCO4A1-AS1 and SLCO4A1 expression in CRC. The expression of SLCO4A1 mRNA and SLCO4A1-AS1 in CRC tissues was analyzed by qRT-PCR. **b** The effects of SLCO4A1-AS1 knockout on the expression of SLCO4A1.

## Data Availability

The datasets during and/or analyzed during the current study are available from the corresponding author on reasonable request.

## Abbreviations

CRC: Colorectal cancer
lncRNAs: Long noncoding RNAs
SLCO4A1-AS1: SLCO4A1 antisense RNA 1
miRNAs: MicroRNAs
NCTs: Noncancerous tissues
ATCC: American Type Culture Collection
siRNAs: Small interfering RNAs
cDNA: Complementary DNA
qRT-PCR: Quantitative RT-PCR
BSP: Bisulfite sequencing PCR
MSP: Methylation-specific PCR
CCK8: Cell Counting Kit 8
PI: Propidium iodide
co-IP: Co-immunoprecipitation
TCGA: The Cancer Genome Atlas
CCLE: Cancer Cell Line Encyclopedia
GSEA: Gene set enrichment analysis
DEGs: Differential expression genes
GO: Gene Ontology
KEGG: Kyoto Encyclopedia of Genes and Genomes
FISH: Fluorescent in situ hybridization
IHC: Immunohistochemistry
ROC: Receiver operating characteristic
OS: Overall survival
DFS: Disease-free survival
AUC: Area under curve
CHX: Cycloheximide
ceRNA: Competing endogenous RNA

## References

[1] Sung H, Ferlay J, Siegel RL, Laversanne M, Soerjomataram I, Jemal A, Bray F (2021). Global cancer statistics 2020: GLOBOCAN estimates of incidence and mortality worldwide for 36 cancers in 185 countries. CA..

[2] Lichtenstein P, Holm NV, Verkasalo PK, Iliadou A, Kaprio J, Koskenvuo M, Pukkala E, Skytthe A, Hemminki K (2000). Environmental and heritable factors in the causation of cancer–analyses of cohorts of twins from Sweden, Denmark, and Finland. N Engl J Med.

[3] Augoff K, McCue B, Plow EF, Sossey-Alaoui K (2012). miR-31 and its host gene lncRNA LOC554202 are regulated by promoter hypermethylation in triple-negative breast cancer. Mol Cancer.

[4] Xiang JF, Yin QF, Chen T, Zhang Y, Zhang XO, Wu Z, Zhang S, Wang HB, Ge J, Lu X (2014). Human colorectal cancer-specific CCAT1-L lncRNA regulates long-range chromatin interactions at the MYC locus. Cell Res.

[5] Qingyu SK (2016). LncRNAs: the ideal composer of the melody for life. Biomed Sci..

[6] Zhu Y, Chen P, Gao Y, Ta N, Zhang Y, Cai J, Zhao Y, Liu S, Zheng J (2018). MEG3 activated by vitamin D inhibits colorectal cancer cells proliferation and migration via regulating clusterin. EBioMedicine.

[7] Yan T, Shen C, Jiang P, Yu C, Guo F, Tian X, Zhu X, Lu S, Han B, Zhong M (2021). Risk SNP-induced lncRNA-SLCC1 drives colorectal cancer through activating glycolysis signaling. Signal Transduct Target Ther.

[8] Hu X, Xing W, Zhao R, Tan Y, Wu X, Ao L, Li Z, Yao M, Yuan M, Guo W (2020). HDAC2 inhibits EMT-mediated cancer metastasis by downregulating the long noncoding RNA H19 in colorectal cancer. J Exp Clin Cancer Res.

[9] Bian Z, Zhang J, Li M, Feng Y, Wang X, Zhang J, Yao S, Jin G, Du J, Han W (2018). LncRNA–FEZF1-AS1 promotes tumor proliferation and metastasis in colorectal cancer by regulating PKM2 Signaling. Clin Cancer Res.

[10] Bian Z, Jin L, Zhang J, Yin Y, Quan C, Hu Y, Feng Y, Liu H, Fei B, Mao Y (2016). LncRNA-UCA1 enhances cell proliferation and 5-fluorouracil resistance in colorectal cancer by inhibiting miR-204-5p. Sci Rep.

[11] Bian Z, Zhang J, Li M, Feng Y, Yao S, Song M, Qi X, Fei B, Yin Y, Hua D (2017). Long non-coding RNA LINC00152 promotes cell proliferation, metastasis, and confers 5-FU resistance in colorectal cancer by inhibiting miR-139-5p. Oncogenesis.

[12] Zhou M, Bian Z, Liu B, Zhang Y, Cao Y, Cui K, Sun S, Li J, Zhang J, Wang X (2021). Long noncoding RNA MCM3AP-AS1 enhances cell proliferation and metastasis in colorectal cancer by regulating miR-193a-5p/SENP1. Cancer Med..

[13] Liu B, Liu Y, Zhou M, Yao S, Bian Z, Liu D, Fei B, Yin Y, Huang Z (2020). Comprehensive ceRNA network analysis and experimental studies identify an IGF2-AS/miR-150/IGF2 regulatory axis in colorectal cancer. Pathol Res Pract..

[14] Li M, Bian Z, Jin G, Zhang J, Yao S, Feng Y, Wang X, Yin Y, Fei B, You Q (2019). LncRNA-SNHG15 enhances cell proliferation in colorectal cancer by inhibiting miR-338-3p. Cancer Med.

[15] Liu Y, Liu B, Jin G, Zhang J, Wang X, Feng Y, Bian Z, Fei B, Yin Y, Huang Z (2019). An integrated three-long non-coding RNA signature predicts prognosis in colorectal cancer patients. Front Oncol.

[16] Wei Y, Wei L, Li J, Ma Z, Zhang Q, Han Z, Li S (2020). SLCO4A1-AS1 promotes cell growth and induces resistance in lung adenocarcinoma by modulating miR-4701–5p/NFE2L1 axis to activate WNT pathway. Cancer Med..

[17] Wu K, Xu T, Song X, Shen J, Zheng S, Zhang L, Tao G, Jiang B (2021). LncRNA SLCO4A1-AS1 modulates colon cancer stem cell properties by binding to miR-150–3p and positively regulating SLCO4A1. Lab Invest..

[18] Li Q, Jiang B, Qi Y, Zhang H, Ma H (2020). Long non-coding RNA SLCO4A1-AS1 drives the progression of non-small-cell lung cancer by modulating miR-223-3p/IKKα/NF-κB signaling. Cancer Biol Ther.

[19] Wang Z, Jin J (2019). LncRNA SLCO4A1-AS1 promotes colorectal cancer cell proliferation by enhancing autophagy via miR-508-3p/PARD3 axis. Aging (Albany NY).

[20] Tang R, Chen J, Tang M, Liao Z, Zhou L, Jiang J, Hu Y, Liao Q, Xiong W, Tang Y (2019). LncRNA SLCO4A1-AS1 predicts poor prognosis and promotes proliferation and metastasis via the EGFR/MAPK pathway in colorectal cancer. Int J Biol Sci.

[21] Yu J, Han Z, Sun Z, Wang Y, Zheng M, Song C (2018). LncRNA SLCO4A1-AS1 facilitates growth and metastasis of colorectal cancer through β-catenin-dependent Wnt pathway. J Exp Clin Cancer Res.

[22] Huang Z, Wen P, Kong R, Cheng H, Zhang B, Quan C, Bian Z, Chen M, Zhang Z, Chen X (2015). USP 33 mediates S lit-R obo signaling in inhibiting colorectal cancer cell migration. Int J Cancer.

[23] Takahashi Y: Co-immunoprecipitation from Transfected Cells. In: *Protein-Protein Interactions: Methods and Applications.* Edited by Meyerkord CL, Fu H. New York, NY: Springer New York; 2015: 381–389. 10.1007/978-1-4939-2425-7_25 25859964

[24] Subramanian A, Tamayo P, Mootha VK, Mukherjee S, Ebert BL, Gillette MA, Paulovich A, Pomeroy SL, Golub TR, Lander ES (2005). Gene set enrichment analysis: a knowledge-based approach for interpreting genome-wide expression profiles. Proc Natl Acad Sci USA.

[25] Yu G, Wang LG, Han Y, He QY (2012). clusterProfiler: an R package for comparing biological themes among gene clusters. OMICS.

[26] Ehrlich M (2009). DNA Hypomethylation in cancer cells. Epigenomics.

[27] Schopf FH, Biebl MM, Buchner J (2017). The HSP90 chaperone machinery. Nat Rev Mol Cell Biol.

[28] Liu Y, Baek J, Zhang H, Diez R, Cole R, Semenza G (2007). RACK1 competes with HSP90 for binding to HIF-1alpha and is required for O(2)-independent and HSP90 inhibitor-induced degradation of HIF-1alpha. Mol Cell.

[29] Cai L, Li L, Chen X, Jia L (2021). Regulation of SKP2 protein stability by heat shock protein 90 chaperone machinery. Signal Transduct Target Ther.

[30] Prince T, Sun L, Matts RL (2005). Cdk2: A Genuine Protein Kinase Client of Hsp90 and Cdc37. Biochemistry.

[31] Hydbring P, Bahram F, Su Y, Tronnersjo S, Hogstrand K, von der Lehr N, Sharifi HR, Lilischkis R, Hein N, Wu S (2010). Phosphorylation by Cdk2 is required for Myc to repress Ras-induced senescence in cotransformation. Proc Natl Acad Sci USA.

[32] Sears RC, Nuckolls F, Haura E, Taya Y, Tamai K, Nevins JR (2000). Multiple Ras-dependent phosphorylation pathways regulate Myc protein stability. Genes Dev.

[33] Yang Y, Wang F, Huang H, Zhang Y, Xie H, Men T (2019). lncRNA SLCO4A1-AS1 promotes growth and invasion of bladder cancer through sponging miR-335-5p to upregulate OCT4. Onco Targets Ther.

[34] Zhang J, Li Z, Liu L, Wang Q, Li S, Chen D, Hu Z, Yu T, Ding J, Li J (2018). Long noncoding RNA TSLNC8 is a tumor suppressor that inactivates the interleukin-6/STAT3 signaling pathway. Hepatology.

[35] Zhang F, Mao D, He Z, Li W, Zhang X, Li L (2021). SLCO4A1-AS1 regulates laryngeal squamous cell carcinoma cell phenotypes via the Wnt pathway. Oral Dis..

[36] Yang Z, Xu F, Wang H, Teschendorff AE, Xie F, He Y (2021). Pan-cancer characterization of long non-coding RNA and DNA methylation mediated transcriptional dysregulation. EBioMedicine..

[37] Mao J, Gao W, Xue L, Wang J, Zhao L (2021). The lncRNA SLCO4A1-AS1/miR-876–3p/RBBP6 axis regulates cell proliferation and apoptosis in acute lymphocytic leukemia via the JNK signaling pathway. Int J Lab Hematol..

[38] Tadesse S, Anshabo AT, Portman N, Lim E, Tilley W, Caldon CE, Wang S (2020). Targeting CDK2 in cancer: challenges and opportunities for therapy. Drug Discov Today.

[39] Pashtan I, Tsutsumi S, Wang S, Xu W, Neckers L (2008). Targeting Hsp90 prevents escape of breast cancer cells from tyrosine kinase inhibition. Cell Cycle.

[40] Dang Chi V (2012). MYC on the path to cancer. Cell.

