# Supplementary figures and images for: SLCO4A1-AS1 promotes colorectal tumourigenesis by regulating Cdk2/c-Myc signalling
Source: J Biomed Sci. 2022 Jan 17;29:4. doi: 10.1186/s12929-022-00789-z (PMC8762969; doi:10.1186/s12929-022-00789-z)

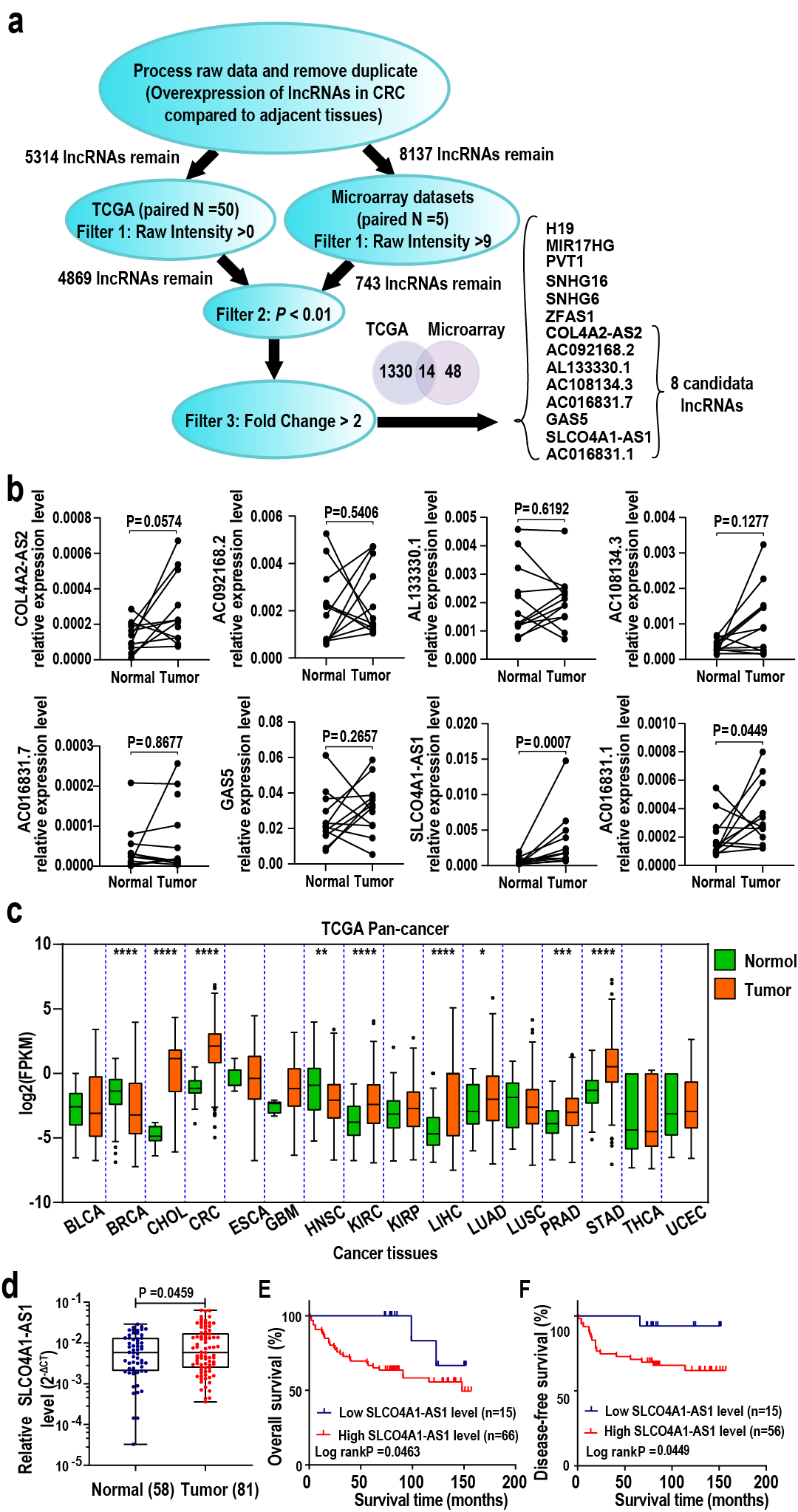

Supplement: Supplementary file 4 — Additional file 4: Fig. S1. SLCO4A1-AS1 is upregulated in CRC and predicts poor prognosis. a The flow chart for selected candidate lncRNAs overexpressed in CRC compared to adjacent tissues from TCGA and our previous microarray datasets. b The expression of eight candidate lncRNAs was quantified in 12 paired CRC tissues and their adjacent tissues. c SLCO4A1-AS1 was overexpressed in multiple cancer types from TCGA databases. d Relative expression levels of SLCO4A1-AS1 were quantified by qRT-PCR in CRC cohort 2. e and f Overall survival (e) and Disease-free survival (f) according to SLCO4A1-AS1 levels were analyzed by Kaplan–Meier method in CRC cohort 2. *P < 0.05; **P < 0.01; ***P < 0.001. [file 12929_2022_789_MOESM4_ESM.png]

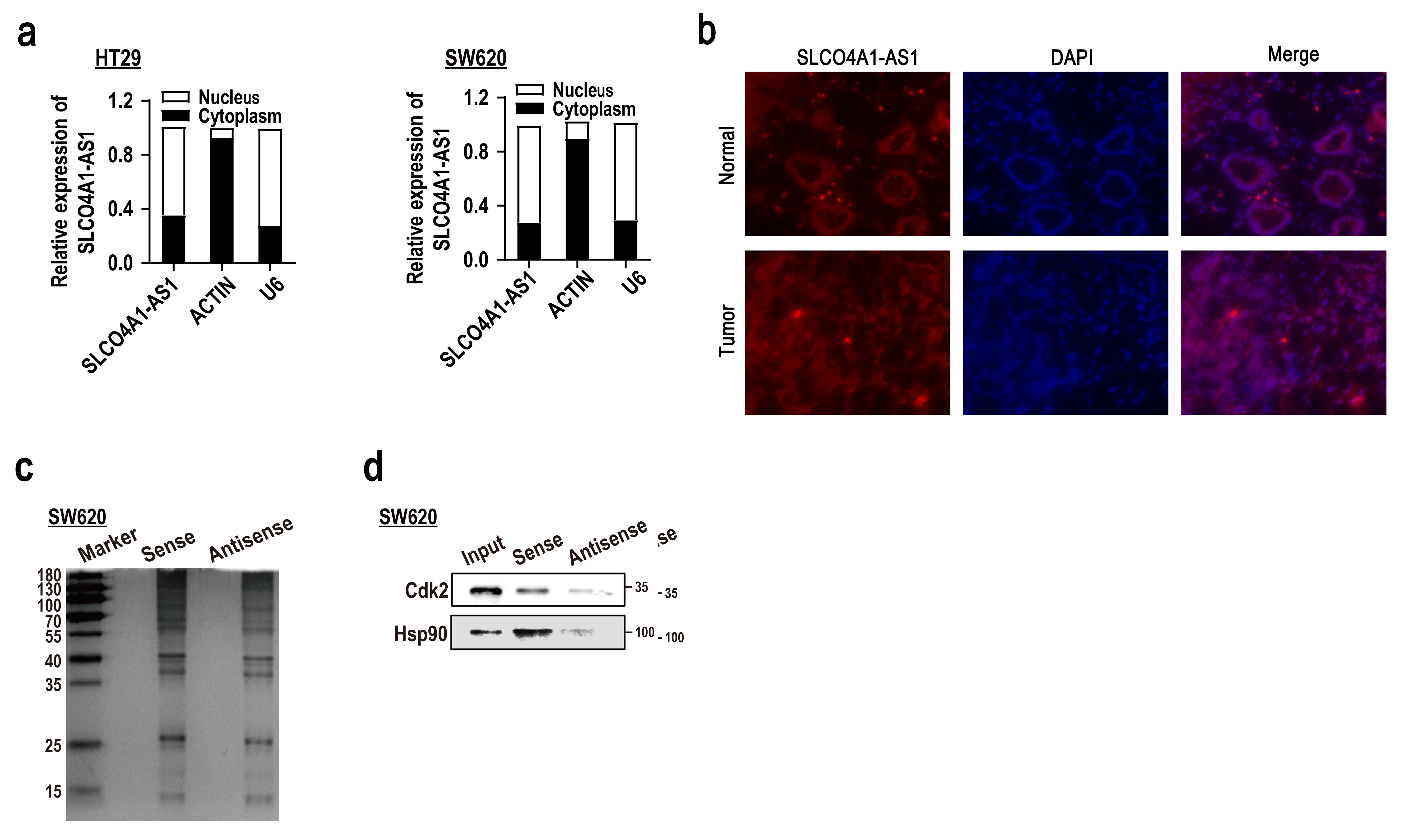

Supplement: Supplementary file 6 — Additional file 6: Fig. S2. Subcellular localization and binding proteins of SLCO4A1-AS1 in CRC. a Subcellular localization of SLCO4A1-AS1 was determined by qRT-PCR in HT29 and SW620 cells b Subcellular localization of SLCO4A1-AS1 was determined by FISH in CRC tissues c Proteins retrieved from the SLCO4A1-AS1 pull-down assay were analyzed by SDS-PAGE in SW620 cells. d Western blotting analyses of Hsp90 and Cdk2 using the proteins retrieved from the SLCO4A1-AS1 pull-down assay in SW620 cells. *P < 0.05; **P < 0.01; ***P < 0.001. [file 12929_2022_789_MOESM6_ESM.png]

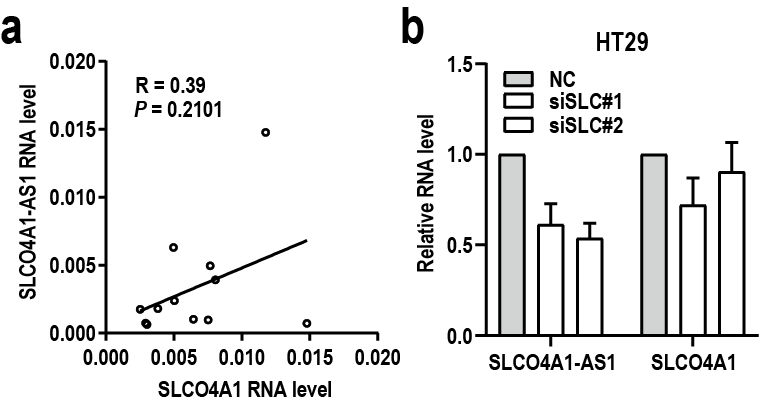

Supplement: Supplementary file 9 — Additional file 9: Fig. S3. The relationship between SLCO4A1-AS1 and SLCO4A1 in CRC. a Correlation analysis of SLCO4A1-AS1 and SLCO4A1 expression in CRC. The expression of SLCO4A1 mRNA and SLCO4A1-AS1 in CRC tissues was analyzed by qRT-PCR. b The effects of SLCO4A1-AS1 knockout on the expression of SLCO4A1. [file 12929_2022_789_MOESM9_ESM.png]
